# The Role of Total Small Bowel Length and its Measurement in Metabolic Bariatric Surgery. A Systematic Review

**DOI:** 10.1007/s11695-026-08715-0

**Published:** 2026-05-07

**Authors:** Niccolo Petrucciani, Chiara Cuzzocrea, Margherita Floris, Marta Zerunian, Nunzio Velotti, Paolo Aurello, Marta Goglia, Maria Vittoria Mascolini, Mirto Foletto, Chiara Giulia Fontanella, Mario Musella, Gianfranco Silecchia

**Affiliations:** 1 Department of Medical and Surgical Sciences and Translational Medicine, Faculty of Medicine and Psychology, St. Andrea Hospital, Sapienza University of Rome, Rome, Italy; 2 Division of Digestive Surgery, Clinique des Cedres, Ramsay Santé, Cornebarrieu, France; 3 Department of Advanced Biomedical Sciences, University of Naples Federico II, Naples, Italy; 4 Department of Industrial Engineering, University of Padua, Padova, Italy; 5 Week Surgery Unit, Department of Surgical, Oncological and Gastroenterological Sciences, University Hospital of Padova, Padua, Italy, Azienda Ospedaliera di Padova, Padova, Italy

**Keywords:** Obesity, Total small bowel length, Metabolic bariatric surgery, Weight loss, Nutritional deficiencies, Complications

## Abstract

This systematic review evaluated the role and measurement of total small bowel length (TSBL) in metabolic bariatric surgery (MBS) following PRISMA guidelines. Twenty-seven studies involving 6968 adult patients undergoing bariatric surgery with TSBL measurement were included. Outcomes of interest included TSBL, weight loss outcomes, comorbidities improvement/resolution, and the relationship between TSBL and weight loss outcomes. Mean TSBL ranged from 506.5 cm to 795.0 cm. Complications related to TSBL measurement occurred in six patients (0.2%), all small bowel perforations. Nutritional deficiencies ranged from 0.3% to 33.8%. In six studies, tailoring procedures according to TSBL was associated with fewer nutritional deficiencies than in controls. Adoption of standardized TSBL measurement protocol may have a notable impact on surgical outcomes with respect to reducing malabsorptive complications in highly malabsorptive procedures.

## Key Points

Measurement of total small bowel length (TSBL) is not a usual practice.

In the present review mean TSBL ranged from 506.5 cm to 795.0 cm.

Lowest and highest values of TSBL were 195 cm and 1220 cm, respectively.

Tailoring the procedure according to TSBL is associated with fewer nutritional deficiencies.

## Introduction

Metabolic Bariatric Surgery (MBS) has proven to be the most effective approach to treat morbid obesity and related comorbidities [1].

Metabolic/bariatric surgery alters the anatomy and physiology of the gastrointestinal tract, specifically affecting nutrient absorption and hormonal production. Several techniques have been developed consisting in a more extensive small intestine bypass, such as the Roux-en-Y gastric bypass (RYGB), the one-anastomosis gastric bypass (OAGB), the single-anastomosis duodenal-ileal switch (SADIS), the biliopancreatic diversion with duodenal switch (BPD-DS), and others [2]. The core of these procedures lies in a reduction in the length of the small intestine, by excluding a variable portion of its proximal segment, generally in association with a reduction in gastric volume [3].

Therefore, the small intestine and its length play a pivotal role in the outcomes of MBS [4]. This extends as well to its complications, particularly in terms of intractable malnutrition and short small bowel syndrome (SBS) [5, 6]. Despite these aspects, systematic measurement of total small bowel length (TSBL) is not a usual practice among metabolic bariatric surgeons. According to the latest data, less than 30% of surgeons routinely measure TSBL [7–10].

The prevailing evidence in the current literature is gathered mostly from non-live human measurements, and it indicates that SBL varies across a wide range of lengths depending on anthropometric factors of the patient [11]. It has also been studied how intraoperative measurement of the TSBL leads to a variety of results among surgeons [12]. Thus, a consistent protocol for intraoperative assessment of TSBL emerges as crucial to elucidate its role in the effectiveness of metabolic bariatric surgery [13]. 

Hence, the aim of this review is to systematically assess the current literature on the role of TSBL and its measurement in the evolution of metabolic bariatric surgery. Building on this premise, we will investigate the intraoperative and preoperative measurement techniques currently employed. Accordingly, we intend to assess the correlations, if observed, between TSBL and the anthropometric and clinical characteristics of patients. Consequently, our focus will also extend to the relationship between TSBL and surgical outcomes, as well as complications. Through this comprehensive analysis, the review ultimately seeks to suggest how small intestine length might be leveraged to enhance and personalize interventions, striving for an evolution toward a surgical approach that is both effective and safe.

## Methods

### Study Design

A systematic review was conducted in keeping with the PRISMA guidelines [14]. The study was registered on the PROSPERO database. The included population consisted of subjects ≥ 18 years old who has undergone elective metabolic bariatric surgery with TSBL measurement, intraoperatively or preoperatively. Outcomes of interest included TSBL, weight loss outcomes, comorbidities improvement/resolution, and the relationship between TSBL and weight loss outcomes. We defined our study eligibility using the populations-interventions-comparators-outcomes study design (PICO) framework:

- Population: Adult patients with obesity undergoing metabolic bariatric surgery;
- Interventions: Metabolic bariatric surgery, including TSBL measurement;
- Outcomes: TSBL in obese patients; weight loss outcomes and comorbidities improvement/resolution; relationship between TSBL and weight loss outcomes.

### Search Strategies

An electronic search of MEDLINE, Scopus, and Cochrane Library (Wiley) databases was performed on September 31st 2025, looking for relevant studies that could be included in this study. The search was performed by setting the following terms: “small bowel length”, “total small bowel length”, “obesity”, “weight loss surgery”, “bariatric surgery”.

The same searches were carried out without using the function [MeSH terms] in order to extract the larger potential number of relevant articles. The Boolean operator “AND” was used to combine parts of the subject terms, and “OR” was used to expand the search.

Two independent reviewers (CC and MF) screened titles and abstracts, assessed full-text versions, and extracted data. Disagreements were resolved by re-extraction or third-party adjudication (NP).

### Data Extraction and Quality Assessment

The literature search was performed by two independent reviewers (CC and MF) using a predefined search strategy. No automation tools were used in the process. Duplicate studies were removed manually. Each reviewer then independently examined the titles, abstracts, and/or full texts of included manuscripts to ensure that all inclusion criteria were met before extracting the following data: [1] first author’s name, [2] year of publication, [3] study design, [4] country of origin, [5] number of patients included, [6] number of patients undergoing metabolic bariatric surgery with total small bowel length measurement, patients’ characteristics [7], type and characteristics of the surgical procedures [8], total small bowel length and length of the common channel [9], outcomes after metabolic bariatric surgery including weight loss outcomes [10], incidence of malnutrition [11], rate of nonresponders [12], and comorbidities improvement/resolution [13], relationship between total small bowel length and outcomes after metabolic bariatric surgery [14]. Missing or unclear information was not taken into consideration.

### Inclusion and Exclusion CriteriaIn

Studies evaluating TSBL in patients with obesity undergoing metabolic bariatric surgery, in patients ≥ 18 years of age, that enrolled more than 5 patients, were included. Studies meeting any of the following exclusion criteria were excluded from the present review: [1] non-English studies, [2] animal studies, [3] abstracts, [4] review articles, [5] case reports or case series including less than 5 subjects; [6] editorials or letters, [7] studies not including TSBL measurement; [8] patient age < 18; [9] studies published in the literature prior to the year 2000. Where overlapping registries were identified or suspected, the more recent study was included for analysis.

### Primary and Secondary Outcomes

The primary outcome of the present study was to estimate the TSBL in male and female patients with obesity undergoing metabolic bariatric surgery.

Secondary outcomes included assessment of the common channel length, weight loss outcomes, incidence of malnutrition, incidence of nonresponders to metabolic bariatric surgery, comorbidities improvement/resolution; relationship between TSBL and common channel length and weight loss outcomes; correlation between TSBL and anthropometric factors.

The collected patient’s factors were age, gender, preoperative body mass index (BMI), comorbidities, type of surgery, and postoperative outcomes. Primary outcome was defined at the time of the first studies’ selection, while secondary outcomes were included following title and abstract review in order to capture a complete and accurate representation of the patient and surgical characteristics that have been evaluated by current literature.

### Risk of Bias Assessment

The Risk of Bias In Non-randomized Studies of Interventions (ROBINS-I) tool was used to rate risk of bias for non-randomized included studies [15]. This tool assesses seven domains: risk of bias from confounding, selection of participants, classification of interventions, deviations from intended interventions, missing data, measurement of outcomes, and selection of the reported results. A proposed judgment about the risk of bias arising from each domain is generated by an algorithm, based on answers to the signaling questions. Judgment can be “Low”, “Moderate”, and “Serious” risk of bias.

Version 2 of the Cochrane risk-of-bias tool was used for randomized trials (RoB 2) [16]. RoB 2 is structured into a fixed set of domains of bias, focusing on different aspects of trial design, conduct, and reporting. Within each domain, a series of questions (‘signaling questions’) aims to elicit information about features of the trial that are relevant to risk of bias. A proposed judgment about the risk of bias arising from each domain is generated by an algorithm, based on answers to the signaling questions. Judgement can be ‘Low’ or ‘High’ risk of bias or can express ‘Some concerns’.

Two reviewers independently assessed each study (CC, MF), and disagreements were resolved by third-party adjudication (NP). No automation tools were used.

### Statistical Analysis

Patients’ characteristics and outcomes were summarized and described as means +- SD or medians (range) for continuous variables or percentages for categorical variables. The results were fully tabulated.

## Results

### Study Selection and Risk of Bias Assessment

**Fig. 1:**
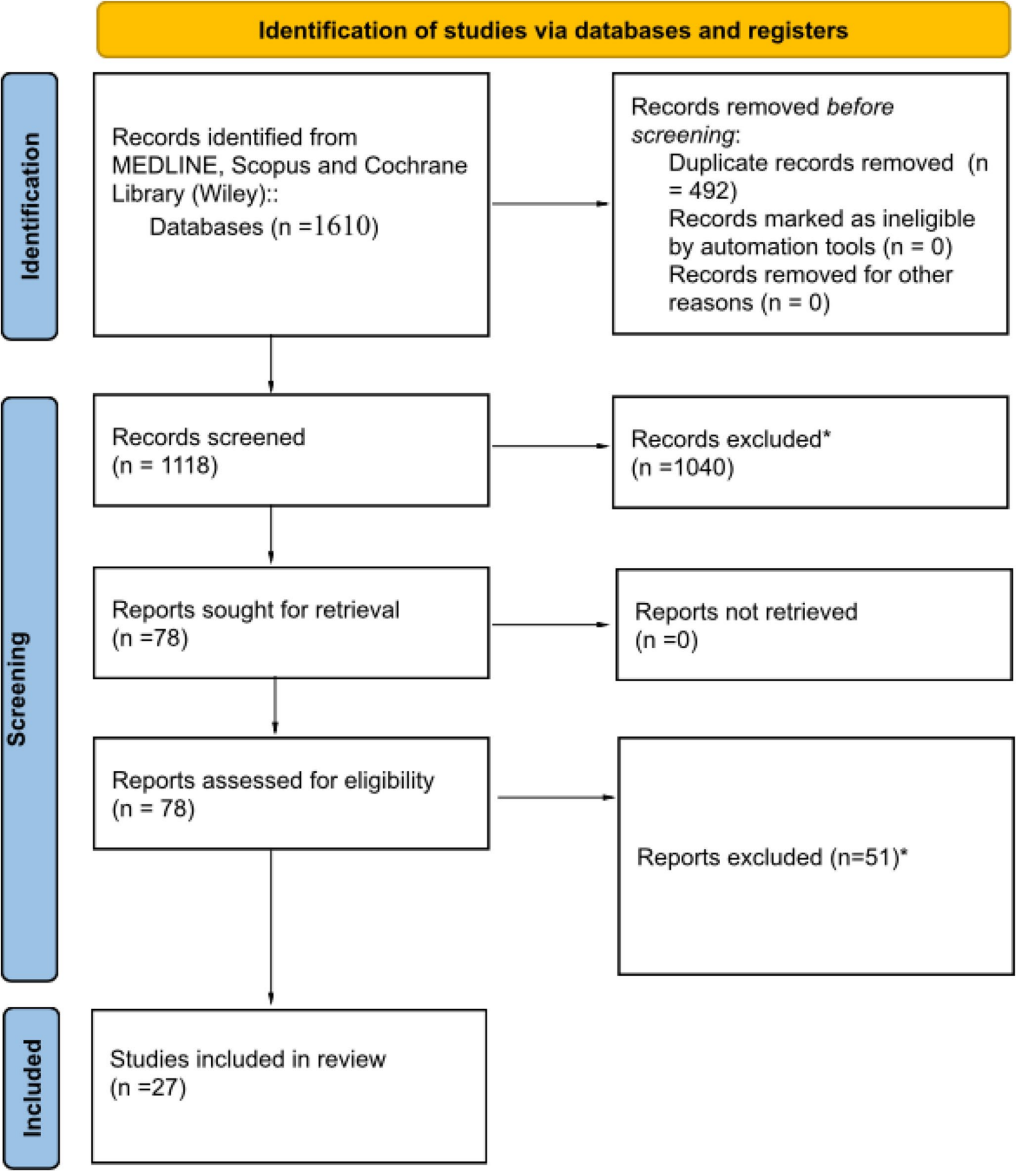
PRISMA flow diagram

**Fig. 2:**
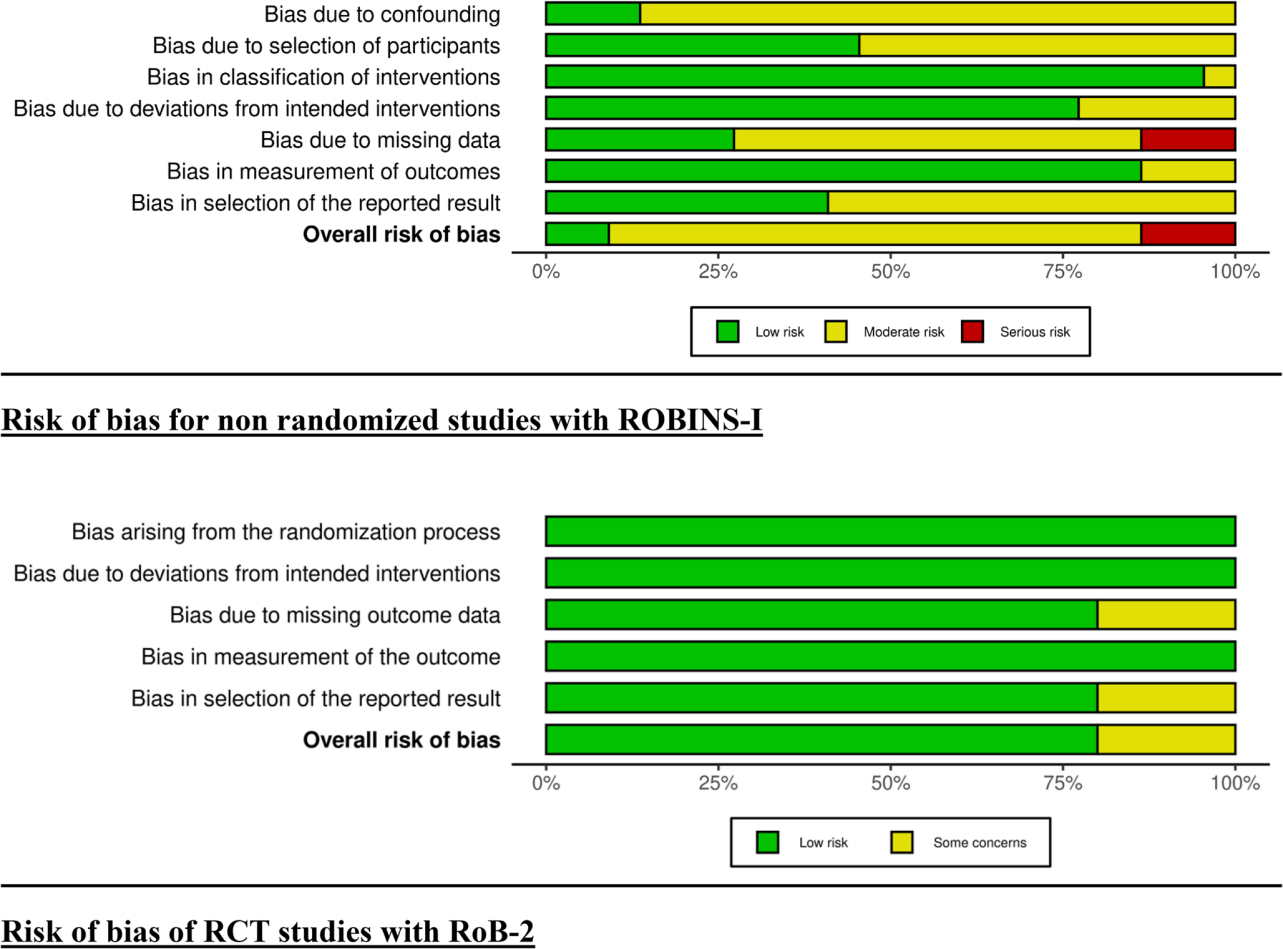
Risk of bias in the included studies

The systematic search process is depicted in Fig. 1. Twenty-seven articles evaluating TSBL in adult patients with obesity undergoing metabolic bariatric surgery were included. The selected studies were published between 2004 and 2025 and included a total of 6968 patients accrued between 2002 and 2022 (Table 1). Most of the included studies were single-institution, retrospective reports. Five studies were randomized controlled trials. Eighteen studies had a control group. Target follow-up (FU) was 1 year in the majority of studies, with four reports with FU > 5 years. Most studies were conducted in European countries or the United States, alongside five articles from Eastern countries and one from Egypt. Figure 2 reports the risk of bias assessment. The majority of non-randomized studies were classified as “moderate”, with the main concerns related to the risk of bias in the selection of the reported results, the risk related to missing data, and the risk associated with deviations from the intended interventions. As shown in Table 2, the assessment of randomized studies has revealed an overall low risk of bias.

**Table 1:** Characteristics of the included studies

Author	Country	Study type	*N*	F/M	BMI (mean ± SD)	Control group	Mean Follow-up
Abellan et al.	Spain	Prospective	151	79/36 22/14	42.2 ± 4.1	1	1 year
Ahmed et al.	USA	Retrospective	547	427/120	46.3*	0	< 7 years
Albaugh et al.	USA	Observational	208	171/33	46.9 (35.0-72.8)	0	2 years
Almalki et al.	Taiwan	Retrospective	620	386/234	38.9 ± 7.6	0	1 year
Athanasiadis et al.	USA	Retrospective	30	30/0	< 50% Bypassed bowel group :47.3 ± 11.6 > 50% Bypassed bowel group : 50 ± 8.6	1	2 years
Estrada et al.	USA	Retrospective	61	55/6	42.59 (39–47)	NR	1 year
Fair et al.	USA	Retrospective	119	35/24 37/23	^#^Pre-TBL group: 51.8 ± 8.8 Post-TBL group: 54.6 ± 13.1	1	1 year
Gadiot et al.	The Netherlands	RCT	444	370/74	^##^VLRL-LRYGB: 42.7 ± 4.5 S-LRYGB 42.3 ± 4.4	1	5 years
Hernández Martínez et al.	Spain	Prospective observational	565	NR	(41–66) *	1	1 year
Hosseini et al.	Iran	Retrospective observational	162	117/21 7/17	^φ^SASI group: 45.35 ± 4.6 SASJ group: 45.19 ± 3.8	1	1 year
Käkelä et al.	Finland	Prospective	70	55/15	41.9 ± 4.3	0	1 year
Karimi Behnagh et al.	Iran	Retrospective observational	961	745 /216	44.32 ± 5.96	0	18 months
Komaei et al.	Italy	Retrospective observational	64	26/6 25/7	44.2 ± 5.8	1	1 year
Liu et al.	China	Prospective	148	61/87	44.94 ± 10.58	1	1 year
Malo et al.	Canada	RCT	20	9/1	^φφ^ LADS group: 46.1 ± 1.2 BPD/DS group: 45.8 ± 2.2	1	2 years
McConnell et al.	USA	Retrospective	124	66/58	^χ^ Group 1 (150 cm) : 53.9 Group 2 (100 cm) : 54.25 Group 3 (90 –80 cm) : 60	1	4 years
Miras et al.	UK	RCT	53	53/0	^χχ^ L-RYGB group: 43 ± 8 S-RYGB group: 42 ± 6	1	1 year
Nabil et al.	Egypt	RCT	60	50/10	^τ^ Conventional group: 52.2 ± 9.7 Distal group: 54.9 ± 9.2	1	1 year
Navez et al.	Belgium	Prospective	90	57/33	44.8 ± 5.5	1	1 year
Ruiz-Tovar et al.	Spain	Retrospective observational	300	240/60	41.3 ± 8.2	1	5 years
Savassi-Rocha et al.	Brazil	Prospective observational	100	76/24	46.29 ± 6.20	0	1 year
Shah et al.	Norway	Retrospective	187	122/65	^ττ^ Group 1: 54.6 Group 2: 58.5 Group 3: 57.4	1	10 years
Slagter et al.	The Netherlands	RCT	212	187/25	40.8 ± 3.7	1	1 year
Soong et al.	Taiwan	Retrospective observational	940	586/354	^ζ^ Group I: 40.6 ± 7.7 Group II: 39.8 ± 7.4	1	1 year
Tacchino	Italy	Observational	443	342/101	NR	0	NR
Våge et al.	Norway	Retrospective observational	182	20/15 80/67	^ζζ^ Group A: 50.6 ± 1.3 Group B: 52.1 ± 0.6	1	2 years
Valera Mora et al.	Italy	Prospective	107	85/22	48.9 ± 8.8 (F) 48.1 ± 6.1 (M)	0	2 years

N., number of included patients; F, females; Male, males; BMI, body mass index; SD, standard deviation; RCT, randomized controlled trial

* Data reported as median (range)

^#^ Pre-TBL: Before the implementation of a TBL measurement protocol; post-TBL: after the implementation of a TBL measurement protocol

^##^ Very long Roux limb laparoscopic Roux- en-Y gastric bypass (VLRL-LRYGB): fixed 100-cm CC and variable length RL, fixed 60-cm BPL; standard laparoscopic Roux-en-Y gastric bypass (S-LRYGB): fixed 150- cm RL and variable length CC, fixed 60-cm BPL

^φ^ Single anastomosis sleeve jejunal (SASJ); Single anastomosis sleeve ileal (SASI) bypass surgery

^φφ^ LADS: Long alimentary limb duodenal switch; BPD/DS: Biliopancreatic diversion with duodenal switch

^χ^ Three groups based on the CC length: group 1: 150 cm; group 2: 100 cm; group 3: 80–90 cm

^χχ^ L-RYGB: long limb RYGB with 150-cm biliopancreatic limb; S-RYGB: standard limb RYGB with 50-cm biliopancreatic limb

^τ^ Conventional technique: fixed anastomosis 200 cm from the ligament of Treitz; Distal technique: anastomosis 400 cm from the ileocecal valve

^ττ^ Group 1: standard gastric bypass (RYGB) with a Roux limb of 150 cm and biliopancreatic limb of 60 cm; group 2: distal gastric bypass (DRYGB) with a RL of 270 cm, BPL of 200 cm, and common channel of 150 cm; and group 3: DRYGB with RL of 220, BPL of 200 cm, and CL of 200 cm

^ζ^ Group I: OAGB with tailored limb according to preoperative body mass index; group II: CC fixed at 400 cm with implementation of mesurement of TSBL

^ζζ^ Group A: gastric remnant with a volume of approximately 200 ml, an alimentary limb (AL) of 250 cm, and a common channel (CC) of 100 cm; group B: gastric remnant of 100–120 ml, an AL of 40%, and a CC of 10% of the small bowel length

**Table 2:** Surgical procedures and TSBL measurement in the included studies

Author	Surgical procedures	Open/laparoscopic	Technique of TSBL measurement	*N*. of measures	TSBL in cm mean (range)	Complications of TSBL measurement
Abellan et al.	RYGB	Laparoscopic	Marked microforceps	2, 3 if necessary	^#^ Obese group: 567 (335–860) Superobese group:591 (475–780)	NR
Ahmed et al.	RYGB	Open/laparoscopic	Sum of BPL, AL and CC Measurements made through different means (grasper, string, ruler) by different surgeons	1	600 (509–710)	NR
Albaugh et al.	RYGB	Laparoscopic	Total length of the small intestine from the Ligament of Treitz to the cecum was measured using identical laparoscopic bowel graspers with 10 cm markings. At the time of surgery, 10 cm segments of unstretched, small bowel were measured, and the total small bowel length (SBL) was recorded.	2 (in 95% of cases)	592 (390–910)	59 patients excluded because of adhesions or mesenteric tethering causing difficulties during measurement; In 94 patients, time of measurement was recorded with an average measurement time of 2 min and 48 s; No adverse events related to the measurement process
Almalki et al.	SG	Laparoscopic	NR	1	739.8 ± 115.7 (380–1050)	2 small bowel perforation: 1 repaired immediately 1 required laparotomy two days later
Athanasiadis et al.	RYGB	Laparoscopic	NR	NR	TALL: 590 (400–1075) BPL: 40 (15–90)	NR
Estrada et al.	RYGB	Laparoscopic	NR	1	580 (550–640)	Median operative time: 102 min (IQR 79–119 min)
Fair et al.	SADI-S	Robotic	Marked robotic arms, on the antimesenteric borders	1	CC ^##^ Pre-TBL group: 268.6 Post-TBL group: 309.8	NR
Gadiot et al.	VLRL-LRYGB S-LRYGB	Laparoscopic	NR	1	^υ^ VLRL-LRYGB: 587 (390–890) S-LRYGB: 598 (355–985)	NR
Hernández Martínez et al.	RYGB	Laparoscopic	half stretched method	1	(380–820)	NR
Hosseini et al.	SASI SASJ	Laparoscopic	Marked graspers	1	744 ± 2 31	0
Käkelä et al.	RYGB	Laparoscopic	Sum of BPL, AL and CC Measurements made with marked laparoscopic Babcock instrument on the mesenteric border	1	743.5 ± 113.6 (530–1060)	NR
Karimi Behnagh et al.	RYGB OAGB	Laparoscopic	Nontraumatizing graded graspers	2	748.9 ± 126.4 (195.0–1220.0)	1 intestinal rupture
Komaei et al.	Tailored OAGB	Laparoscopic	Atraumatic laparoscopic forceps marked at 10 cm, on the antimesenteric border, with fully stretched bowel	1	625.6 ± 110.5 (410–930)	0
Liu et al.	RYGB	Laparoscopic	The length of the jejunum- ileum to the ileocecal region was measured from the Treitz ligament along the mesenteric wall with a 25-cm inelastic cloth.	1	714.44 (430–1005)	NR
Malo et al.	LADS BPD/DS	Laparoscopic	NR	NR	^υυ^ LADS group: 769.5 ± 96.9 BPD/DS group: 795.0 ± 66.1	NR
McConnell et al.	BPD BPD/DS	Open	Modest stretch	1	NR	NR
Miras et al.	RYGB Long RYGB	Laparoscopic	Marked laparoscopic graspers, along the mesenteric border	1	^*^ L-RYGB group: 610 (520–910) S-RYGB group: 615 (320–740)	NR
Nabil et al.	OAGB	Laparoscopic	NR	1	^**^Conventional group: 715 ± 123 (600–950) Distal group: 728 ± 103 620–1000	0
Navez et al.	RYGB	Laparoscopic (3 converted to open)	NR	1	585 ± 94.6 (380–815)	NR
Ruiz-Tovar et al.	OAGB	Laparoscopic	NR	1	506.5 ± 23.2 (430–600)	Few cases of bowel lacerations in the serosa were sutured with absorbable single stitches. No cases of unadverted bowel lacerations.
Savassi-Rocha et al.	RYGB	Open	The intestinal limbs were measured on the anti-mesenteric margin, applying the minimum tension necessary to obtain a straight measurement of the limbs.	1	671.4 ± 115.7 (434–990)	The average time spent measuring the intestine was 8.7 ± 2.1 min (5.0 to 15.0 min).
Shah et al.	RYGB DRYGB	Laparoscopic	NR	1	(420–870)	Mean surgical time was longer in the DRYGB groups
Slagter et al.	OAGB	Laparoscopic	Normal position and without applying traction.	NR	^&^Standard BP length: 644.8 ± 124.8 (355–1020) Tailored BP length 669.5 ± 130.6 (295–990)	1 small bowel perforation
Soong et al.	Tailored OAGB	Laparoscopic	NR	NR	744.9 ± 119.3 (400–1100)	2 iatrogen intestine perforation immediately sutured
Tacchino	General surgery including bariatric procedures.	Open and laparoscopic	One measurement performed with no tension applied and the second with fully stretched intestine.	2	690 ± 93.7 (350–1049).	NR
Våge et al.	BPD/DS	Open	Moistened umbilical tape on semistretched bowel	1	NR	0
Valera Mora et al.	BPD	Open	NR	NR	671 ± 99 (460–1050)	NR

N., number; TSBL, total small bowel length; RYGB, Roux-en-Y gastric bypass; NR, not reported; BPL, Biliopancreatic limb; AL, alimentary limb; CC, common channel; SG, sleeve gastrectomy; TALL, total alimentary limb length; OAGB, one anastomosis gastric bypass; SADIS, single anastomosis duodeno-ileal switch; SASI, Single anastomosis sleeve ileal bypass surgery; SASJ, Single anastomosis sleeve jejunal gastric bypass; LADS, Long alimentary limb duodenal switch; BPD/DS, Biliopancreatic diversion with duodenal switch; BPD, bilipopancreatic diversion; DRYGB, Distal RYGB;

^#^ Obese group: patients with BMI 35–50 kg/m^2^; Superobese group: patients with BMI > 50 kg/m^2^

^##^ Pre-TBL: Before the implementation of a TBL measurement protocol; post-TBL: after the implementation of a TBL measurement protocol

^υ^ Very long Roux limb laparoscopic Roux- en-Y gastric bypass (VLRL-LRYGB): fixed 100-cm CC and variable length RL, fixed 60-cm BPL; standard laparoscopic Roux-en-Y gastric bypass (S-LRYGB): fixed 150- cm RL and variable length CC, fixed 60-cm BPL

^υυ^ LADS: Long alimentary limb duodenal switch; BPD/DS: Biliopancreatic diversion with duodenal switch

^*^ L-RYGB: long limb RYGB with 150-cm biliopancreatic limb; S-RYGB: standard limb RYGB with 50-cm biliopancreatic limb

^**^ Conventional technique: fixed anastomosis 200 cm from the ligament of Treitz; Distal technique: anastomosis 400 cm from the ileocecal valve

^&^ In the standard group: BP limb length of 150 cm; tailored BPL group: the BP limb length was based on TSBL using the following three predefined categories: first, for a TSBL shorter than 500 cm, a BP limb length of 150 cm was used; second, for a TSBL ranging between 500 and 700 cm, a BP limb length of 180 cm was used; and third, for a TSBL longer than 700 cm, a BP limb length of 210 cm was used

### Patients’ Characteristics and Surgical Procedures

Overall, 6968 patients in 27 studies were included in the systematic review. In the 26 studies reporting the information, 4621 patients were females and 1768 males. The mean preoperative BMI ranged from 38.9 kg/m2 to 60 kg/m^2^. In the 24 studies reporting the information, surgical procedures included: Sleeve gastrectomy in 620 patients, RYGB in 2654, OAGB in 1576, SADI-S in 119, SASI in 138, SASJ in 24, BPD in 433. Postoperative morbidity ranged from 0.2% to 23%. The more frequent complications were anastomotic leakages and bleedings, wound infection, thrombotic events, diarrhea, and anemia.

### Total Small Bowel Length Measurement

**Fig. 3:**
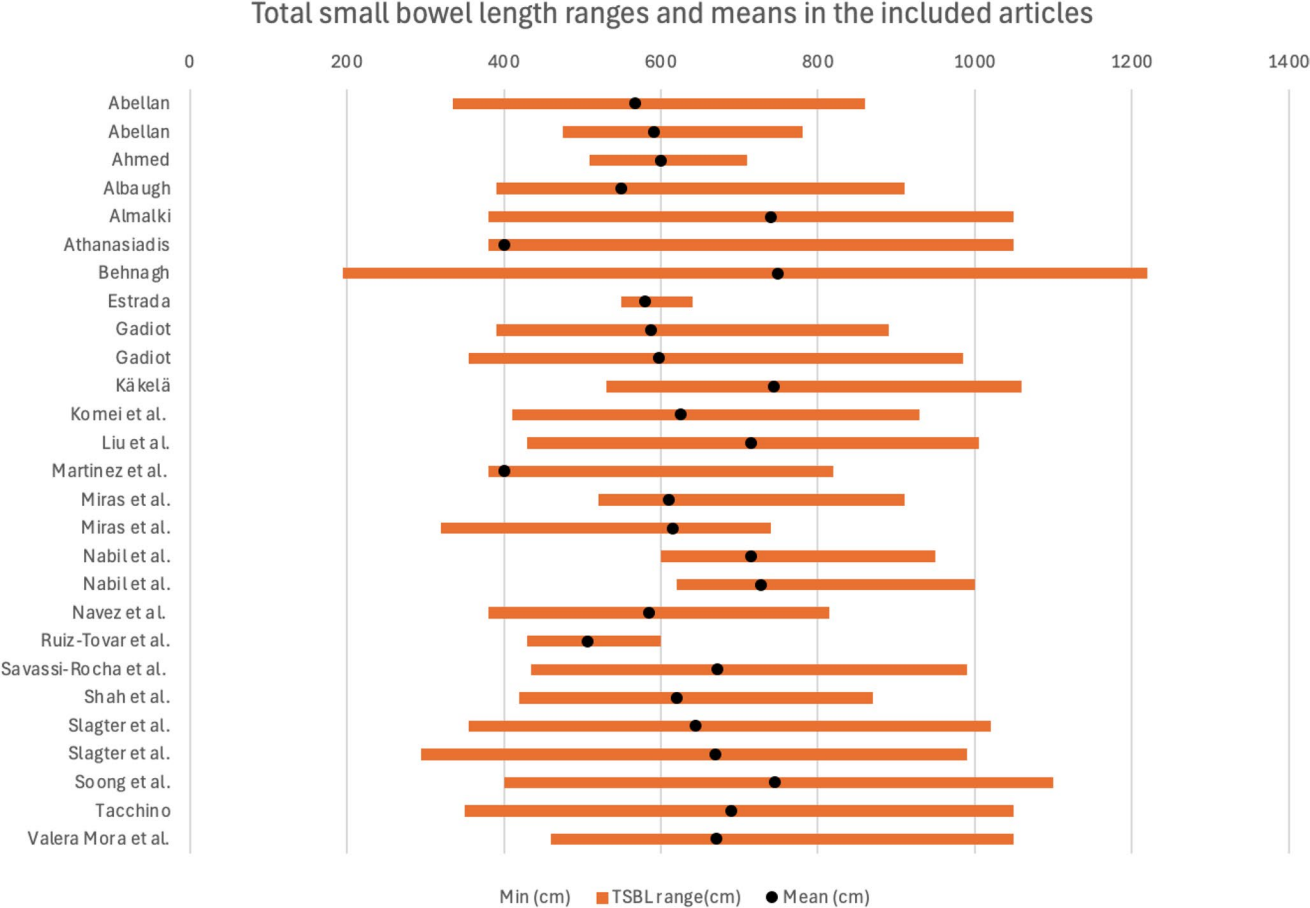
Total small bowel length ranges and means in the included articles

The TSBL was measured in all the included studies (Table 2). The surgical technique to measure the small bowel was reported in 16 studies and, in most cases, consisted in measurement with marked graspers (5–10 cm), from the ligament of Treitz to the ileocecal valve. In 3 studies, two or more TSBL measurements were performed. Mean TSBL ranged from 506.5 cm to 795.0 cm in the included studies. The lowest observed value was 195 cm, and the highest was 1220 cm. Figure 3 shows the TSBL ranges in the included studies. Six studies mention either the operative time or the time employed for TSBL measurement [17–22].

From the studies reporting the information, complications related to TSBL measurements occurred in 6 patients and consisted in small bowel perforation [18, 20, 23, 24]. Patients were treated as follows: 5 cases were immediately repaired with sutures [18, 20, 23, 24], 1 case required laparotomy on postoperative day one [23].

### Postoperative Outcomes and Relationship with TSBL

Postoperative weight loss was reported in twenty-four studies. At 1 y FU, mean %EWL ranged from 51.9 to 91.1 (Table 3). Comorbidities resolution was reported by eleven studies, mainly focusing on remission of type 2 diabetes (T2D), arterial hypertension (HTN), and hyperlipidemia.

**Table 3:** Correlation between TSBL and anthropometric and clinical variables

Author	Anthropometric and biochemical factors related to TSBL	1-year weight loss	Malnutrition (%) and nutritional deficiencies	Comorbidities resolution	Other relevant findings
Abellan et al.	NR	EWL% 71.4 ± 14.9% (Obese group) 65.7 ± 15.3% (Superobese group) ^ψ^	Ferritin deficiency: 7.4% (CL < 50%) 1.2% (CL > 50%) Iron deficiency 14.7% (CL < 50%) 1.2% (CL > 50%) Albumin deficiency 2.9% (CL < 50%) 2.4% (CL > 50%) Total proteins deficiency 25.6% (CL < 50%) 14.8% (CL > 50%)	NR	TSBL had no relation with EWL%, nor did the %CC
Ahmed et al.	NR	-35.1 (weight, % of pre-surgery, median)	NR	Improvement in: T2DM: 82.4% Hyperlididemia: 79.4% Hypertension: 73.7%	Limb lengths were not associated to outcomes Pre-operative BMI was not related to TSBL
Albaughet al.	NR	No detectable effects of CCL or TALL on weight loss up to 24 months	NR	NR	NR
Almalki et al.	Height Weight Waist circumference Serum levels of LDL Hemoglobin C-peptide Glycated hemoglobin C (A1C) Gamma-glutamyl transferase (r-GT)	TWL % 28.6 ± 7.0 (SBL < 600 cm group) 30.9 ± 9.4 (SBL 600–800 cm group) 31.8 ± 7.4 (SBL > 800 cm group)	Anemia 14.4% (SBL < 600 cm group) 12.8% (SBL 600–800 cm group) 6.7% (SBL > 800 cm group) Hypoalbuminemia 0.3% (SBL < 600 cm group) 1.3% (SBL 600–800 cm group) 0.9% (SBL > 800 cm group) Secondary hyperparathyroidism 0.3% (SBL < 600 cm group) 31.0% (SBL 600–800 cm group) 20.0% (SBL > 800 cm group)	A1C% 5.2 ± 0.4% (SBL < 600 cm group) 5.2 ± 0.4% (SBL 600–800 cm group) 5.3% ± 0.2 (SBL > 800 cm group)	Multivariate analysis confirmed that body height and A1C% could independently predict small bowel length
Athaniasiadis et al.	NR	EBIL% after 1 year: 64% (> 50% bypass) vs. 37.3% (≤ 50%)	23% complications (ulcers, DVT, SBO, 1 death); Vitamin A deficiency 20–22%; Vitamin D 3–7%. No reintervention for malnutrition	T2DM remission 100%, HTN 67%, GERD 73%	NR
Estrada et al.	NR	At 1 year, the EWL (%) was 51.9 (40–66)	3 severe complications from the surgery while no severe cases of malnutrition were encountered	Yes, improvement or resolution of OSAS, T2DM, HTN, HLD	NR
Fair et al.	NR	EWL %/Mean (SD) Pre-TBL group: 52/71.4 (18.4) Post-TBL group: 41/65.8 (26) ^#^ TWL %/ Mean (SD) Pre-TBL group: 52/34.9 (7.7) Post-TBL group: 41/32.0 (9.3)	Hypoalbuminemia: Pre-TBL group: 26.3% Post-TBL group: 8.3%	NR	NR
Gadiot et al.	NR	At 5 years of follow-up (FU), a signifcant diference in %TWL and %EWL in favor of VLRL-LRYGB group was found. %EWL 70,89 (LRYGB) 79,82 (VLRL-LRYGB) %TWL 28,57 (LRYGB) 32,15 (VLRL-LRYGB) ^##^	Overall complication rate during the 3-year follow-up (> 30 days after surgery) was 15.8% in the VLRL-LRYGB group versus 9% in the S-LRYGB group Vitamin B12 deficiency was higher in S-LRYGB group than in VLRL-LRYGB group (7.1 vs. 2.0%, *p* = 0.013).	VLRL-RYGB group had higher percentage of resolution of type 2 diabetes when compared to patients in the S-LRYGB group (75.0% vs. 51.1%)	NR
Hernández Martínez et al.	Sex BMI	EWL% 70.1	17.3% chronic anemia	NR	NR
Hosseini et al.	NR	EWL% 87.88 ± 23.33% (SASI group) 76.79 ± 18.51% (SASJ group) TWL% 38.72 ± 10.36% (SASI group) 33.78 ± 7.73% (SASJ group)^φ^	Vitamin D3 deficiency 25.3% (SASI group) 25% (SASJ group)	T2D 84.6% (SASI group) 83.3% (SASJ group) HTN 79% (SASI group) 50% (SASJ group) Hyperlipidemia 76.9% (SASI group) 70% (SASJ group OSAS 87.9% (SASI group) 87.5% (SASJ group NAFLD 90% (SASI group) 68.7% (SASJ group 8/138 of SASI group were revised to SASJ for excessive weight loss (EWL > 100%)	Longer common limb was associated with less %EWL at 6 months. As well as fewer complications, while larger anastomosis size were associated with higher WL and greater HTN improvement Shorter common channel was significantly correlated with reversal operation
Käkelä et al.	Serum TG, ALT and steatosis were associated to TSBL at baseline Serum TG remained associated a 1 year follow up	BMI (Kg/m²) 32.6 ± 5.0 (CC < 520 cm) 31.5 ± 4.2 (CC 520–600 cm) 31.8 ± 4.0 (CC > 600 cm)	NR	Hyperlipidemia 70%	NR
Karimi Behnagh et al.	Height: most significant Age Weight	NR	NR	NR	No correlations with clinical conditions were found
Komaei et a.	NR	EWL% 66.2 ± 17.1% (Fixed-BPL group) 63.3 ± 13.7% (Tailored-BPL group) TWL% 33.4 ± 7.9% (Fixed-BPL group) 33.3 ± 6.6% (Tailored-BPL) ^ψψ^	Vitamin A deficiency 31.2% (Fixed-BPL group) 9.4% (Tailored-BPL group) Vitamin D3 deficiency 28.1% (Fixed-BPL group) 6.2% (Tailored-BPL group) Hypoalbuminemia 31.2% (Fixed-BPL group) 9.4% (Tailored-BPL group)	NR	NR
Liu et al.	Sex Females: 656.80 ± 92.98 Males: 754.81 ± 86.17 Height	EWL% RL/BPL = 3: 34.3 RL/BPL = 3.5 27.88 RL/BPL = 4: 18.50 ^ε^	NR	T2DM Remission (%) RL/BPL = 3: 27.4 RL/BPL = 3.5 29.77 RL/BPL = 4: 27.40	NR
Malo et al.	NR	EWL%* 81.6 ± 6.6% (LADS group) 97.1 ± 11.1% (BPD/DS group) TWL%* 40.9 ± 3.7% % (LADS group) 48.3 ± 4.9% (BPD/DS group) BMI 27.2 ± 1.5 kg/m2 (LADS group) 23.8 ± 2.7 kg/m2 (BPD/DS group) ^φφ^	2 mild cases at 12 months in the BPD/DS group	mean glycated hemoglobin* 0.04 ± 0.01 (LADS group) 0.04 ± 0.004 (BPD/DS group) mean cholesterol ratio* 2.5 ± 0.7 (LADS group) 2.4 ± 0.6 (BPD/DS group)	Mean calcium, vitamin D, hemoglobin, zinc, and copper levels were statistically lower in the BPD/DS group than in the LADS group. *
McConnell et al.	NR	Weight loss (Kg) 150-cm CC group: 45; 100-cm CC group: 55.8; 80- to 90-cm CC group: 61.5. Mean %EWL 150-cm CC group: 44; 100-cm CC group: 57.3; 80- to 90-cm CC group: 54.8.	Albumin (g/dl) 150-cm CC group: 4.0; 100-cm CC group: 3.9; 80- to 90-cm CC group: 3.6. Vitamin D (ng/ml) 150-cm CC group: 36; 100-cm CC group: 19; 80- to 90-cm CC group: 19.	NR	NR
Miras et al.	NR	TWL% 29 ± 8% (Long limb group) 30 ± 8% (Standard limb group) ^χχ^	NR	T2DM remission 77% (Long limb group) 62% (Standard limb group)	The long limb RYGB did not produce any measurable difference in fasting or post-prandial GLP-1 concentration, time to peak concentration, and total GLP-1 AUC
Nabil et al.	Weight (*r* = 0.474) and height (*r* = 0.314).	EBWL(%)= Conventional group: 63.1 ± 8.7 Distal group: 69.4 ± 15.4 ^τ^	Nutritional deficiencies at 1 year Hemoglobin deficiency conventional group: 26.7% distal group: 66.7% Iron deficiency conventional group: 23.3% distal group: 76.7% Calcium deficiency conventional group: 6.7% distal group: 6.7% Hypoalbuminemia conventional group:6.7% distal group:36.7%	Comorbidities remission rates T2DM Conventional group (64.3%) Distal group (62.5%) Hypertension Conventional group (40%) Distal group 66.7% Hyperlipidemia Conventional group 82.6% Distal group 85.7%	NR
Navez et al.	Height Sex	EBMIL(%) = 71.1 ± 17.7	NR	NR	NR
Ruiz-Tovar et al.	No significant correlation between TBL and BMI, gender or any other anthropometric variable, could be observed.	WL (Kg) = 40.8 ± 7 EWL (%) = 91.1 ± 10.9 TWL (%) = 36.1 ± 4.6	Iron deficiency anemia (1%)	Total remission rate of T2DM at 1, 2 and 5 years postoperatively was 93.3%, 95.2% and 95.2%, respectively Hypertension remission rate at 1, 2 and 5 years was 88.4%, 85.3% and 81.4%, respectively Dyslipidemia remission rate was 98.3% at 1, 2 and 5 years.	BPL length and BPL/TBL ratio directly correlated with UBMIL, while CL length and CL/TBL ratio showed an inverse correlation. Moreover, CL/TBL ratio proved to be the most accurate predictor of weight loss
Savassi-Rocha et al.	Sex Height	WL (kg) 45.5 SD 12.1 median 44.5 WL (%) 35.8 SD 6.7 median 36.6 EWL (%) 71.3 SD 14.3 median 69.9	NR	NR	NR
Shah et al.	NR	Yes, better EWL in groups with shorter TALL. %EWL at 10 year follow-up was 75.7 and %TWL 42.3.	Increased incidence of malnutrition and higher risk of internal hernia when CL = 150 cm if compared with the standard RYGB^ττ^	NR	NR
Slagter et al.	NR	^χ^ Standard group EWL% 87.0 (22.5) TWL % 32.8 (6.9) Tailored group EWL% 89.0 (20.3) TWL% 33.1 (6.2)	Standard BP limb length Anaemia: 13.5% Iron deficiency: 8.7% Hypoalbuminemia: 29.8% Vitamin A deficiency: 8.7% Vitamin B12 deficiency: 4.8% Vitamin D deficiency:6.7% Tailored BP limb length based on TSBL: Anaemia: 13.3% Iron deficiency: 10.5% Hypoalbuminemia: 30.5% Vitamin A deficiency: 8.6% Vitamin B12 deficiency: 5.7% Vitamin D deficiency: 14.3%	Total remission: T2DM standard group: 67% tailored group: 86% HTN standard group: 43% tailored group: 60% OSAS standard group: 81% tailored group: 72%	NR
Soong et al.	NR	EWL% 77.0 ± 32.2% (Group I) 83.3 ± 51.5% (Group II) ^ζ^ TWL% 32.0 ± 8.5% (Group I) 34.0 ± 8.4% (Group II)	Anemia 11.1% (Group I) 5.9% (Group II) Secondary Hyperparathyroidism 33.8% (Group I) 21.7% (Group II) Hypoalbuminemia 2.8% (Group I) 1.5% (Group II)	T2DM 84.7% (Group I) 84.1% (Group II)	NR
Tacchino	Sex: men had a longer small bowel than women (729 ‡ 85 versus 678 ‡ 92, *P* < 0.0001) Weight: SBL showed no significant correlation to body weight Height: height was positively correlated with SBL (*r* = 0.32, *P* < 0.0001).	NR	NR	NR	NR
Våge et al.	NR	EWL% 62.0 ± 2.8% (Group A) 79.1 ± 1.2% (Group B) ^ζζ^	Protein malnutrition 1 (Group A) 5 (Group B) Hyperparathyroidism 9 (Group A) 20 (Group B) Vitamin D deficiency 13 (Group A) 22 (Group B)	NR	An inverse relationship between BMI and serum level of vitamin D was found, both preoperative and post-operative
Valera Mora et al.	NR	Fat mass (kg) after BPD 22 ± 14 (women) 28 ± 16 (man) *	NR	T2DM 88%	Length of the excluded intestine was found to be not predictive of weight loss at 2 years

*Data refers to outcome results at 2 years follow-up

TSBL, total small bowel length; NR, not reported; EWL%, percentage of excess weight loss; T2DM, type 2 diabetes mellitus; BMI, body mass index; TWL%, percentage of total weight loss; LDL, low density lipoprotein; EBIL, excess body mass index loss; DVT, deep vein thrombosis; SBO, small bowel occlusion; HTN, hypertension; GERD, gastroesophageal reflux disease; OSAS, obstructive sleep apnoea syndrome; NAFLD, non-alcoholic fatty liver disorder; TG, triglycerides; ALT, alanine aminotransferase; GLP-1, glucagon-like peptide 1; EBWL, excess body weight loss

^ψ^ Obese group: patients with BMI 35–50 kg/m^2^; Superobese group: patients with BMI > 50 kg/m^2^

^#^ Pre-TBL: Before the implementation of a TBL measurement protocol; post-TBL: after the implementation of a TBL measurement protocol

^##^ Very long Roux limb laparoscopic Roux- en-Y gastric bypass (VLRL-LRYGB): fixed 100-cm CC and variable length RL, fixed 60-cm BPL; standard laparoscopic Roux-en-Y gastric bypass (S-LRYGB): fixed 150- cm RL and variable length CC, fixed 60-cm BPL

^φ^ Single anastomosis sleeve jejunal (SASJ); Single anastomosis sleeve ileal (SASI) bypass surgery

^ψψ^ Fixed BPL: OAGB-MGB with a fixed 200-cm BPL; Tailored BPL: OAGB-MGB with a tailored BPL length relative to SBL

^ε^ Roux Limb (RL)/Biliopancreatic limb (BPL) was divided into three groups. The first group: BPL = 50 cm, RL = 150 cm; the second group: BPL = 50 cm, RL = 175 cm; the third group: BPL = 50 cm, RL = 200 cm. The proportional relationship was RL/BPL = 3, RL/BPL = 3.5, RL/BPL = 4

^φφ^ LADS: Long alimentary limb duodenal switch; BPD/DS: Biliopancreatic diversion with duodenal switch

^χχ^ L-RYGB: long limb RYGB with 150-cm biliopancreatic limb; S-RYGB: standard limb RYGB with 50-cm biliopancreatic limb

^τ^ Conventional technique: fixed anastomosis 200 cm from the ligament of Treitz; Distal technique: anastomosis 400 cm from the ileocecal valve

^ττ^ Group 1: standard gastric bypass (RYGB) with a Roux limb of 150 cm and biliopancreatic limb of 60 cm; group 2: distal gastric bypass (DRYGB) with a RL of 270 cm, BPL of 200 cm, and common channel of 150 cm; and group 3: DRYGB with RL of 220, BPL of 200 cm, and CL of 200 cm

^χ^ In the standard group: BP limb length of 150 cm; tailored BPL group: the BP limb length was based on TSBL using the following three predefined categories: first, for a TSBL shorter than 500 cm, a BP limb length of 150 cm was used; second, for a TSBL ranging between 500 and 700 cm, a BP limb length of 180 cm was used; and third, for a TSBL longer than 700 cm, a BP limb length of 210 cm was used

^ζ^ Group I: OAGB with tailored limb according to preoperative body mass index; group II: CC fixed at 400 cm with implementation of mesurement of TSBL

^ζζ^ Group A: gastric remnant with a volume of approximately 200 ml, an alimentary limb (AL) of 250 cm, and a common channel (CC) of 100 cm; group B: gastric remnant of 100–120 ml, an AL of 40%, and a CC of 10% of the small bowel length

Nutritional deficiencies were observed in a range of 0,3% to 33.8%, primarily represented by hypoalbuminemia, total protein deficiency, iron and ferritin depletion, anemia, secondary hypoparathyroidism, and multiple vitamin deficiencies. Patients with nutritional deficiencies were treated mainly conservatively, with few cases requiring surgical revision [20, 22, 25, 26].

### Variables Associated with TSBL and the Utility of TSBL Measurement

Ten studies investigated possible anthropometric or biochemical factors [13, 19, 23, 24, 27–32]. Six studies found correlations between TSBL and height [13, 19, 23, 24, 29, 31], while other anthropometric factors were linked to TSBL in five studies (see Table 3) [13, 23, 24, 27, 30]. Biochemical correlations were observed in two studies [23, 28].

Furthermore, tailoring the procedure according to the TSBL was associated with fewer nutritional deficiencies compared to the control group in six studies [18, 20, 22, 26, 32, 33]. Concurrently, six studies reported lower rates of malnutrition associated with longer common limb length [20, 22, 34–36].

Pertaining to weight loss, most of the studies did not observe significant differences when analyzing different subgroups, whether in the context of a tailored strategy or when comparing different predefined limb lengths [20, 22, 28, 30, 31, 33, 37–39]. In contrast, differences between the groups in terms of %EWL were not infrequently observed, mainly in relation to the length of the common channel [18, 25, 26, 34–36, 40, 41].

Other relevant findings include the identification of predictors of TSBL. Within this framework, anthropometric factors have received the greatest attention [13, 19, 23, 24, 27, 29–32]. Overall, two studies evaluated the presence of correlation between baseline biochemical features and TSBL [23, 28], with notable findings regarding baseline lipid profile, as well as hepatic steatosis [28].

Furthermore, four studies specifically evaluated the relationship between individual limb lengths themselves as well as the TSBL, such as the common channel, the alimentary limb, or the biliopancreatic limb [29, 32, 33, 41].

## Discussion

The impact of TSBL measurement during metabolic bariatric surgery is still debated, and even if TSBL is considered an important factor by many authors, in clinical practice, only a minority of surgeons measure the TSBL or use its value to tailor the surgical procedure [7–10].

Indeed, despite significant variability in small bowel length has been highlighted and investigated in numerous studies over the past decades [11, 13, 19, 42–44], metabolic bariatric surgeons still rely on standardized techniques with a “standard” measure of the bowel limbs [7, 8, 10, 45]. Several reasons may lead practitioners to decide not to measure the entire length of the small intestine. On one hand, concerns are raised regarding the potential prolongation of operative time, particularly in the case of multiple measurements [20]. Additionally, further handling due to the measurement of the bowel could be perceived as increasing the risk of bowel injury or even perforation [20, 33]. Finally, the lack of a standardized measurement technique. The analysis of the studies included in the present systematic review reveals that only a minority of studies reported complications related to the measurement process, which occurred at very low rates and were managed mostly intraoperatively (see Table 2). Only one patient underwent reoperation for iatrogenic bowel lesion due to TSBL measurement [23] and in 5 cases perforations or lacerations were sutured immediately [18, 20, 23, 24, 32]. Only 0.2% of patients from the studies reporting the information experienced complications related to small bowel measurement, representing a small proportion of the overall study population (Table 2). This data may be biased, for example by the fact that studies that include TSBL measurement may be conducted by surgeons with high experience in TSBL measurement or may suffer from publication bias. Regarding the potential impact on operative time, only a minority of the included studies provided explicit data regarding small bowel measurement and operative time [17–19, 21, 36]. Among these, no clinically relevant prolongation of surgical time was reported. This observation may be attributed to the high level of surgical expertise among the operating teams, particularly in the context of bowel handling within metabolic bariatric procedures [22].

To delve deeper into this aspect, we sought to identify and analyze the measurement techniques employed. In particular, the majority of studies employed marked laparoscopic graspers, assessing the entire length of the small intestine, often at the beginning of the procedure, although in some instances, TSBL was determined by summing the measurements of individual limbs [28, 37].

A critical aspect to be considered in relation to intraoperative laparoscopic measurement is the influence of the intestinal border along which the measurement is performed, but particularly the degree of tissue stretching induced by the procedure itself [32]. As this represents a major source of variability in the measurement outcome, many authors explicitly predefined the extent of stretching they intended to apply in order to standardize the technique. The majority opt to measure under fully or partially stretched conditions [26, 27, 33, 34], whereas only a minority perform measurements with the bowel left unstretched [18, 19]. In relation to this specific issue, Tacchino et al. investigated the role of these two variables by performing measurements midway between the mesenteric and antimesenteric borders, as well as conducting sequential assessments first under minimal tension and subsequently on a fully stretched bowel [13]. Furthermore, the team also compared measurements obtained during laparoscopy with those recorded after conversion to laparotomy. Interestingly, the measurements obtained from fully stretched versus non-stretched bowels demonstrated a mean difference of 137 ± 19 cm (range 72–212 cm), with a highly significant predictability of the stretched length from the unstretched value. Concurrently, the comparison between laparotomy and laparoscopy revealed a mean difference of 32.4 ± 11.4 cm (range 10–58 cm). Given that the measurements obtained by stretching the tissues were consistent with those of cadaver studies present in the literature, Tacchino et al. suggested that in both cases the tonicity of the bowel does not affect the TSBL assessment, and the fully stretched method appears to be more reliable in order to obtain repeatable SBL values [13].

The data gathered from the studies included in the present systematic review confirm the remarkable variability in total small bowel length (TSBL), ranging from 195 to 1220 cm, with reported mean values spanning from 506.5 to 795.0 cm (see Table 2). This variability highlights how standardized procedures may, in fact, lead to potential substantial differences in both the extent of %EWL [32, 34, 41] or affect the incidence of postoperative nutritional deficiencies depending on the length of the small intestine [36, 41]. In particular, patients with markedly short TSBL may experience higher incidence of nutritional complications [20, 30].

These considerations have prompted further investigation not only into the measurement of the small intestine, but also into the potential existence of predictive factors for its length [13, 19, 23, 24, 27–32]. Several anthropometric and clinical parameters have been evaluated in the search for factors associated with TSBL. Among these, height emerges most consistently in the present review, having been repeatedly reported as significantly correlated with TSBL [13, 19, 23, 24, 29–31]. Other parameters, such as sex, weight or BMI, have also been investigated, but statistical relevance has not been described as firmly as in the case of height [13, 19, 23, 24, 29–31]. For instance, with regard to sex, the limitation of this correlation has been attributed to its relationship with height, the parameter showing the stronger correlation. Consequently, sex may not necessarily represent an independent predictor [13]. In terms of BMI, for instance, Abellan et al. did not uncover a statistically significant difference between “morbide obese patients” and “super-obese patients” in terms of TSBL [38].

As already mentioned, some authors have investigated those aspects with the specific intent of identifying predictors of SBL [13, 24]. Tacchino suggests that only height is a potential predictor, while Karimi Behnagh et al. believe this positive association to be of limited clinical applicability [13, 24]. Interestingly, biochemical factors have also been shown to be correlated with TSBL by a few authors. Metabolic parameters such as serum triglycerides and LDL cholesterol or glycated hemoglobin C were shown to be associated with statistical significance. Particularly, the latter was confirmed to independently predict SBL [23] in one study; the analysis of this correlation may deserve further investigations.

A potentially relevant aspect of small bowel prediction or assessment lies in determining on which metabolic bariatric procedure it may have the greatest impact. Fourteen of the included studies focused on RYGB, while six examined its impact in the context of OAGB. Only a minority addressed other procedures, such as BPD or its variations, including SADI-S, SASI, or SASJ, and just one study performed measurements in patients undergoing sleeve gastrectomy. These patterns can largely be explained by statistical considerations: RYGB and OAGB currently represent the most frequently performed procedures among those traditionally categorized as ‘malabsorptive,’ namely those that exert their effect by reducing the length of the intestinal alimentary tract [8, 46]. Consistently, the majority of the included studies addressing the classical BPD procedure tend to be less recent [26, 34, 47]. Similarly, the limited focus on SG cases, despite this being the most widely performed metabolic bariatric operation worldwide, can be justified by the fact that SG does not directly alter the small intestine, but rather acts by only restricting gastric volume.

Consequently, in what ways did the measurement prove to be of value within these interventions?

The reasons that led the authors of the included studies to measure small bowel length are diverse, yet, in most cases, they appear to be unified by the intent to tailor the surgical approach and assess its effects, or by the objective of comparing and evaluating the role of different limb length in terms of outcomes and complications.

Notably, the limb examined in relation to its role differed according to the applied procedure. In RYGB, for example, attention was directed toward variability in both the alimentary limb (AL) and the biliopancreatic limb (BPL). Several of these studies focused on the latter, exploring the impact of adapting it to TSBL. Interestingly, the relationship between BPL and the total alimentary limb length (TALL) has recurrently surfaced: as BPL length increases, with a corresponding reduction in TALL, weight loss outcomes appear to improve [17, 36, 41]. The literature reviewed also highlights the importance of preserving a minimum TALL of 300–400 cm, a threshold regarded as protective against deficiency-related complications [17, 36, 41]. In particular, Gadiot et al. investigated the role of the TALL by focusing instead on the AL [25]. The group observed that patients with a shorter intestine achieved greater weight loss when using a longer RL, compared to patients with a longer intestine. This result supported once again the role of a shorter TALL on RYGB outcomes, alongside the underlying necessity of measuring the TSBL in order to assess these variables.

Nevertheless, some authors also reported no impact of TALL, especially with respect to weight loss, thereby highlighting the complexity of this correlation and the likely involvement of other contributing factors [21, 37].

The BPL has drawn similar attention in the context of OAGB [18, 32, 33]. The literature indicates that the afferent limb exerts a limited impact on weight loss, with a more pronounced effect on deficiencies [18, 20, 32, 33], which nonetheless justifies measuring the intestine in order to construct a tailored limb [33]. This can be explained by the fact that, in the context of the single-anastomosis procedure, the predominant role seems to be played by the remaining common channel [20, 32]. For example, Ruiz-Tovar, through the measurement of TSBL, identified an ideal ratio between CC and TSBL, as well as an optimal length for the CC, corresponding to 0.40–0.47 and 200–220 cm, respectively. Those parameters led to a secure long-term weight loss alongside the resolution of related comorbidities. Based on these findings, the group consistently recommends measuring at least the CC, even if assessment of the entire intestine is not deemed necessary [32]. Similarly, Soong and colleagues emphasized the need to measure TSBL to avoid constructing an excessively short CC [20].

Indeed, the impact of CC length is a consistent finding across multiple procedures. Intuitively, a shorter CC results in a greater proportion of bypassed intestine, leading to correspondingly greater weight loss [19, 34]. Most of the included studies focusing on the role of the CC confirm this finding, reporting better outcomes associated with a shorter common limb [17, 19, 26, 34, 35]. Some authors further emphasize the need to measure the intestine to ensure it is not excessively short [48], thereby avoiding malabsorption-related complications [20, 22, 36–38]. Although cases exist in which no significant effect on surgical outcomes has been observed [18, 21, 28, 31, 37], it clearly emerges how the marked variability in small bowel length and the resulting limb configuration alone justifies, and in certain cases mandates, measurement of the entire small intestine [18, 19, 21, 27, 28, 31, 32]. Sixteen of the twenty-one included studies reporting ranges of small bowel length documented the presence of patients with a TSBL of less than 450 cm [13, 18–21, 23–25, 27, 29, 31–33, 36, 38, 39]. Based on these findings, in highly malabsorbitive procedures, such as RYGB and OAGB with long biliopancreatic limbs, SASI, SADI, and RYGB distalization intestinal measurement of the TSBL or at least the common limb must be mandatory to avoid nutritional complications, whereas standard RYGB and OAGB with 150 cm BPL limb are safer even in the absence of TSBL measurement.

### Limitations

This systematic review has some limitations to be acknowledged. First, the wide heterogeneity of the studies, including the study design, the type of surgical procedure, reported outcomes and tailoring approach limited the possibility of a direct comparison and statistical meta-analysis. Second, most of the included evidence derives from retrospective, single-center studies, with only a limited number of randomized controlled trials (5/27) and relatively short follow-up periods, which may have affected the strength of our conclusions. Future studies should employ standardized measurement protocols and longer follow-up in order to better define the role of total small bowel length in tailoring metabolic bariatric surgery.

## Conclusions

Despite being relatively uncommon, measurement of the small intestine does not appear to substantially increase operative risk or extend operative time, particularly when performed by an experienced surgeon. However, small bowel iatrogenic lesions are possible even in experienced hands. The adoption of a TSBL measurement protocol in metabolic bariatric surgery has the rationale of acquiring awareness of the constructed limb lengths during the procedure and of directly tailoring the surgical technique. Such approaches have demonstrated an impact on surgical outcomes compared to procedures performed without assessment, particularly with respect to reducing malabsorptive complications in highly malabsorbitive procedures, as knowledge of limb length allows for the assurance of an adequate minimal length to prevent malnutrition-related complications, without compromising weight loss outcomes. Particular attention should be paid to more malabsorptive procedures such as RYGB and OAGB with long biliopancreatic limbs, SASI, SADI, and RYGB distalization. In these cases, intestinal measurement of at least the common limb must be mandatory to avoid nutritional complications. Potential predictive factors for TSBL, with height emerging as the predominant variable, may be useful. Concurrently, alternative non-invasive approaches beyond intraoperative measurement, such as radiological techniques, warrant further investigation to establish their feasibility as they may allow tailoring of the procedure avoiding potential small bowel injuries and limiting nutritional complications.

## Data Availability

The data that support the findings of this study are available from the corresponding author, upon reasonable request.
